# Supplementary material for: Clinical bleeding patterns and management techniques of abnormal uterine bleeding at a teaching and referral hospital in Western Kenya
Source: PLoS One. 2020 Dec 2;15(12):e0243166. doi: 10.1371/journal.pone.0243166 (PMC7710065; doi:10.1371/journal.pone.0243166)
Supplement: S1 Appendix — (DOCX) [file pone.0243166.s002.docx]

## Appendix I: Questionnaire

Study Number: ___________

**Patient’s Demographic Data**:

1. Date of birth of patient
2. Level of Education
3. Not Attended Education
4. Primary education
5. Secondary education
6. University/college

3. Employment Status:

1. Employed
2. Self-employed
3. Student
4. Unemployed
5. Place of Residence:
   - - 1. within Eldoret……………..
       2. Outside Eldoret………………
6. What is your current marital status?
   1. Married
   2. Divorced
   3. Separated
   4. Single
   5. Widowed
   6. Would rather not say
7. Phone number………………………………….
8. Other phone number (Next of kin’s) ………………………………

**SECTION B: MEDICAL HISTORY**

1. What’s your Bleeding Pattern?
2. Heavy bleeding (excessive menstrual bleeding)………………………
3. Prolonged bleeding (bleeding more than 8 days)………….......................
4. Frequent bleeding (more than 4 episodes in 90 day period)………….…
5. Infrequent bleeding (1 or 2 episodes in 90 day period)……………........
6. Intermenstrual bleeding (bleeding in between menstrual periods)…
7. Other (specify)…………………..………………...
8. Have you taken any drugs before onset of the bleeding pattern?

1. Yes_______
2. No_____

**SECTION C: DIAGNOSTIC TESTING:**

1. Laboratory Tests

| **Diagnostic test** | **Date** | **Result** |
| --- | --- | --- |
| Pregnancy test |  |  |
| Complete Blood Count |  |  |
| VIA |  |  |
| TSH |  |  |
| Estradiol |  |  |
| Prolactin |  |  |
| Progesterone |  |  |
| FSH |  |  |
| Endometrial biopsy |  |  |
|  |  |  |

1. Medical Imaging

| **Diagnostic test** | **Date** | **Result** |
| --- | --- | --- |
| CT scan |  |  |
| Ultrasound |  |  |
| MRI |  |  |
| Hysterosalpingogram |  |  |
| Hysteroscopy |  |  |
| Other (specify) |  |  |

1. Current diagnosis………………………..

**SECTION D: Management**

1. What treatment (if any) have you received for per vaginal bleeding so far?
2. No treatment
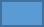

3. Surgery
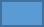

4. Medications
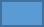

5. Please describe your prior treatments here……………………………….
6. Treated as outpatient and discharged yes
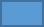
 no
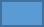

7. Admitted yes
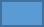
 no
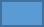

8. Admitted to ICU yes
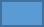
 no
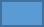

9. Ward:
10. Medical treatment. ……………………
11. Treated by surgery ……………………
12. Died
    1. At Casualty on Arrival
13. In Ward
14. Intra-Op
15. Discharged: Condition at time of discharge-
16. Fully recovered
    1. For clinic follow-up
