# Supplementary material for: Clinical bleeding patterns and management techniques of abnormal uterine bleeding at a teaching and referral hospital in Western Kenya
Source: PLoS One. 2020 Dec 2;15(12):e0243166. doi: 10.1371/journal.pone.0243166 (PMC7710065; doi:10.1371/journal.pone.0243166)
Supplement: Attachment — Submitted filename: Response to Reviewers.docx [file pone.0243166.s003.docx]

24^th^ September 2020

Dear Editor,

**RE:	RESPONSE TO REVIEWERS COMMENTS FOR “CLINICAL BLEEDING PATTERNS AND MANAGEMENT TECHNIQUES OF ABNORMAL UTERINE BLEEDING IN WESTERN KENYA. PLOS ONE”**

***Comment 1: Discussing AUB in Kenya is a matter of interest, however, conclusions based on 108 reported cases give a weak and no representative information for that.***

This study was conducted in a teaching and referral hospital in Western Kenya. The tertiary healthcare facility offers clinical management for abnormal uterine bleeding alongside other gynaecological interventions to women from the greater Western Kenya. This makes the sample representative of the population, however not the whole country. To clarify this further, we have modified the study title to read “**Clinical bleeding patterns and management techniques of abnormal uterine bleeding at a teaching and referral hospital in Western Kenya.**”

***Comment 2: In INTRODUTION, authors should state the interest of the present study, but that information is not clear. For example: the difference between patterns of bleeding and causes of AUB seems confusing.***

This has been modified appropriately. The interest of the study has been highlighted in the second paragraph of the introduction.

The difference between patterns and causes of bleeding have been differentiated as:

*“Clinical bleeding patterns are determined by the heaviness, duration of flow, regularity, and frequency.*

*The causes of AUB can either be structural (PALM) or non-structural (COEIN). Management on the other hand includes the diagnostic techniques and therapeutic interventions offered to the affected women.”*

**Comment 3:	*OBJECTIVES are not well defined. It is not clear what the authors really intent to investigate.***

This has been modified as ***“****This study aimed to determine the clinical bleeding patterns and management of AUB among women in their reproductive age. Specifically, it determined level of adherence to PALM-COEIN diagnostic guidelines for uterine bleeding, diagnostic tests ordered, medical and surgical management offered for women presenting with abnormal uterine bleeding.****”***

**Comment 4:	*In MATERIALS and METHODS is missing important information, like the adopted definitions of AUB patterns and software used for statistical analysis of the data.***

Abnormal uterine bleeding has been defined as: *“an episode of bleeding in a woman of reproductive age,* *who is not pregnant and is of sufficient quantity to require immediate intervention to prevent further blood loss.”* Bleeding Patterns were either heavy, light, frequent, infrequent, regular, irregular, prolonged or short.

Data analysis was conducted using statistical package for social sciences (SPSS) version 22 software.

***Comment 5: In RESULTS, characteristics of the studied population raise some doubts (64.8% had secondary level of education, but only 10.2% have an employment – this should be discussed). In AUB medical management is stated that 78.7% of the patients received antibiotics: this should be discussed.***

These have been discussed as follows:

*“Nearly two-thirds of the study participants had a minimum of secondary education; however only one-tenth of those enrolled were engaged in formal employment. This could be attributed to the low unemployment rates in Kenya especially among women of reproductive age who mainly engage in informal businesses or take care of their families as housewives. This was confirmed by a national survey of Kenyan youth (aged 18-35 years) where 62% of women were unemployed compared to an average unemployment rate of 15% among youths with a minimum of secondary education.”*

*“Because majority of the women presented with intense abdominal pain, they were prescribed for analgesics at the initial stage of treatment alongside antibiotics because of pyrexia and other infection symptoms.*”

***Comment 6: In DISCUSSION, results are compared with those reported with other studies, but there is no explanation about the results obtained by the authors (why analgesics were given to nearly all patients? why myomectomy was performed in 69,9% of the patients? and so on…)***

This has been modified appropriately as follows:

“*Abdominal pain coupled with per vaginal bleeding are the common clinical presentation of abnormal uterine bleeding. Because majority of the women presented with intense abdominal pain, they were prescribed for analgesics at the initial stage of treatment alongside antibiotics because of pyrexia and other infection symptoms.”*

*“Myomectomy was done in more than two-thirds of those who received surgical management due to the fact that fibroids was the most common structural cause of bleeding; its fertility sparing benefit and popularity among the study participants who were women of reproductive age.”*

***Comment 7: In CONCLUSIONS, the only one conclusion of this manuscript is that PALM-COEIN Guidelines are rarely used in Western Kenya, as in many parts of the world.***

The conclusion section has been expanded to include a second conclusion. Furthermore, the study recommendations have also expanded and modified appropriately.

***Other comments:***

i. The manuscript structure has been updated in line with all PLoS requirements.
ii. Dataset has been uploaded for review.
iii. The study’s questionnaire has been appended as additional information.

Sincerely,

**Dr. Godfrey Mutakha** *(Corresponding author)*.
